# Finding Optimal Locations for Implementing Innovative Hypertension Management Approaches Among African American Populations: Mapping Barbershops, Hair Salons, and Community Health Centers

**DOI:** 10.5888/pcd21.230329

**Published:** 2024-02-15

**Authors:** Yui Fujii, Taylor E. Streeter, Linda Schieb, Michele Casper, Hilary K. Wall

**Affiliations:** 1 Division for Heart Disease and Stroke Prevention, National Center for Chronic Disease Prevention and Health Promotion, Centers for Disease Control and Prevention, Atlanta, Georgia; 2 Bizzell US, New Carrollton, Maryland

**Figure:**
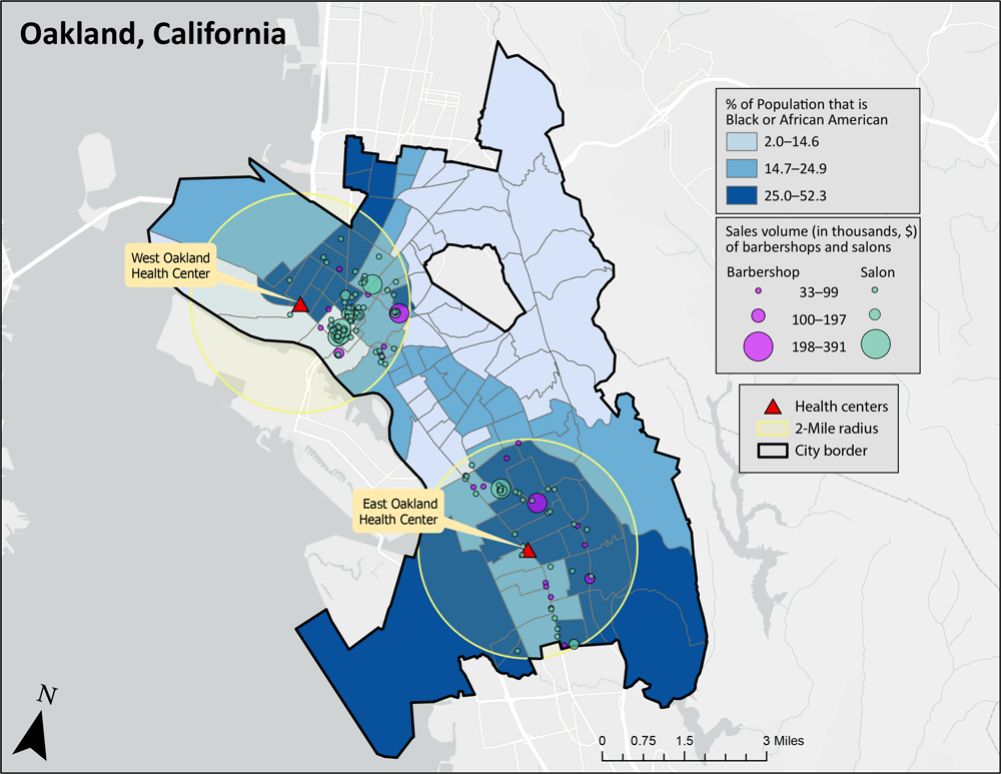
Within a 2-mile radius of the East Oakland Health Center and the West Oakland Health Center, in Oakland, California, numerous barbershops and hair salons are located in census tracts where more than 14.7% of the population is Black or African American. This map provides information for implementing innovative blood pressure management approaches that extend the evidence-based LA barbershop model in African American communities. Sources: US Census Bureau (1), Esri Business Analyst (2).

## Background

An estimated 120 million US adults have hypertension, a leading cause of heart disease, stroke, and kidney disease, and about 93 million of those adults have uncontrolled hypertension (3,4). Black or African American (hereinafter, African American) populations have a disproportionate burden of hypertension and hypertension-related mortality compared with White populations (5,6). The Los Angeles Barbershop Blood Pressure Study, a novel approach to improve hypertension control among African American adults, involved partnerships between local barbershops and pharmacists. Barbers screened African American men for hypertension, and pharmacists provided hypertension medication management. This intervention significantly reduced systolic blood pressure by an average of 21.6 mm Hg among African American men enrolled in the study (7).

The LA barbershop model demonstrated that trusted spaces, like barbershops, can facilitate evidence-based care and contribute to reductions in blood pressure in African American communities (8,9). Although recognized for its impact on hypertension control, this model has not been scaled to real-world settings, nor has it been expanded to include other trusted spaces. Million Hearts, a national initiative to prevent myocardial infarctions, strokes, and other cardiovascular events, is mapping selected communities as part of a feasibility demonstration project to identify patterns of 3 core components needed for large-scale implementation of the LA barbershop model: 1) percentage of the population that is African American, 2) location of community health centers, and 3) location of barbershops and hair salons within a 2-mile radius of community health centers. In this GIS Snapshot, we expand on the LA barbershop model by including hair salons as trusted spaces and focus on these 3 core components in Oakland, California.

## Data and Methods

We obtained data on the percentage of the population that is African American in each census tract in Oakland, California, from the US Census Bureau American Community Survey (2016–2020) (1). We selected the East Oakland Health Center and the West Oakland Health Center because of their interest in partnering in Million Hearts. We used the North American Industry Classification System codes 812111 (barber shops) and 812112 (hair salons) from Esri Business Analyst to obtain geocoded data on barbershops and hair salons, along with their sales volumes (2). We categorized barbershops and hair salons according to tertiles of sales volume.

We created a choropleth map that shows tertiles of the percentage of population that is African American in Oakland (N = 116 census tracts) and drew a 2-mile radius around each health center to capture core components nearby. We included in the map all barbershops (N = 25) and hair salons (N = 112) within a 2-mile radius of each health center; the map also shows these businesses by tertile of sales volume. We used ArcGIS Pro 2.9.2 (Esri) to create the map.

## Highlights

Within a 2-mile radius of both the East Oakland Health Center and the West Oakland Health Center, numerous barbershops and hair salons are located in census tracts in the middle tertile (14.7%–24.9%) and top tertile (25.0%–52.3%) of percentage African American population. More barbershops and hair salons are within a 2-mile radius of the West Oakland Health Center (n = 88) than the East Oakland Health Center (n = 49). However, the East Oakland Health Center has a greater percentage (43%) of shops and salons located in the top tertile of percentage of African American population, compared with those near the West Oakland Health Center (16%). In both locations, there are barbershops and salons in all 3 categories of sales volume. These locations are potential settings for implementing the LA barbershop model of hypertension medication management in real-world settings.

## Action

Million Hearts addresses inequities in health care among racial and ethnic minority populations, including hypertension medication management in trusted spaces for African American people with hypertension. Our map provides valuable information for implementing innovative hypertension management approaches that extend the evidence-based LA barbershop model. Community health centers in Oakland can use this map to identify barbershops and hair salons — trusted spaces in African American communities — to initiate discussions for enhancing community–clinical linkages for hypertension management that are tailored to the needs of African American communities. This asset mapping of 3 core components (percentage African American population, presence of health centers, and nearby barbershops and hair salons) can serve as a model for other communities interested in extending the LA barbershop model of hypertension medication management.

A recent statement from the American Heart Association/American Medical Association (AHA/AMA) indicates that “large-scale implementation and dissemination [of high blood pressure management and control strategies] would help accelerate the translation of evidence-based best practices into care” (10). The AHA/AMA statement is an urgent call for multipronged approaches to optimize blood pressure management in light of the recent national declines in blood pressure control (10). These organizations also recognized the need to increase access to care for populations that have been historically excluded from traditional health care settings and have a disproportionately high burden of hypertension.
